# PCPE2: Expression of multifunctional extracellular glycoprotein associated with diverse cellular functions

**DOI:** 10.1016/j.jlr.2024.100664

**Published:** 2024-10-05

**Authors:** Michael J. Thomas, Hao Xu, Angela Wang, Mirza Ahmar Beg, Mary G. Sorci-Thomas

**Affiliations:** 1 Division of Endocrinology and Molecular Medicine, Department of Pharmacology & Toxicology, Medical College of Wisconsin, Milwaukee, WI, USA; 2 Cardiovascular Research Center, Division of Endocrinology and Molecular Medicine, Medical College of Wisconsin, Milwaukee, WI, USA; 3 Division of Endocrinology and Molecular Medicine, Department of Medicine, Medical College of Wisconsin, Milwaukee, WI, USA

**Keywords:** procollagen C-endopeptidase enhancer 2, PCPE2, *PCOLCE2*, PCPE, *PCOLCE*, cancer, inflammation, lipid metabolism, fibrosis, wound healing, diabetes

## Abstract

Procollagen C-endopeptidase enhancer 2, known as PCPE2 or PCOC2 (gene name, *PCOLCE2*) is a glycoprotein that resides in the extracellular matrix, and is similar in domain organization to PCPE1/PCPE, PCOC1 (*PCOLCE1/PCOLCE*). Due to the many similarities between the two related proteins, PCPE2 has been assumed to have biological functions similar to PCPE. PCPE is a well-established enhancer of procollagen processing activating the enzyme, BMP-1. However, reports show that PCPE2 has a strikingly different tissue expression profile compared to PCPE. With that in mind and given the paucity of published studies on PCPE2, this review examines the current literature citing PCPE2 and its association with specific cell types and signaling pathways. Additionally, this review will present a brief history of PCPE2’s discovery, highlighting structural and functional similarities and differences compared to PCPE. Considering the widespread use of RNA sequencing techniques to examine associations between cell-specific gene expression and disease states, we will show that PCPE2 is repeatedly found as a differentially regulated gene (DEG) significantly associated with a number of cellular processes, well beyond the scope of procollagen fibril processing.

## Structural comparison of procollagen C-endopeptidase enhancer proteins

In 1986 procollagen C-endopeptidase enhancer PCPE (*PCOLCE*) was isolated during its co-purification of type I procollagen C-proteinase from mouse fibroblasts (1, 2, 3). Fourteen years later PCPE2, gene name, *PCOLCE2,* was discovered showing ∼45% amino acid sequence homology to PCPE (4). For clarity, PCPE will be used in this review instead of PCPE1 which is frequently used in the literature to refer to the same protein. Both PCPE and PCPE2 proteins have similar domain structures comprised of 2 N-terminal Complement C1r/C1s, UEGF, BMP-1, or CUB domains, and followed by a linker connected to a C-terminal NETRIN-like (NTR) domain. Typically, CUB domains are structural motifs of approximately 110 residues that coordinate protein-protein binding (5) and are found in extracellular and plasma membrane-associated proteins (5, 6, 7, 8, 9). NTR domains are domains that anchor proteins in the extracellular matrix (ECM) to heparan sulfate proteoglycans (HSPG) (10, 11, 12, 13). Since PCPE was discovered and characterized well before PCPE2, most aspects of PCPE2 function have been inferred from studies on PCPE (14). Recently, however, a published study demonstrates that PCPE2 exhibits inhibitory properties (15) which will be discussed later in this review. Protein structural similarities using Blastp (16) showed that human PCPE and PCPE2 share ∼45.6% homology with a mean % similarity of 80.6 (17), with “similarity” defined as amino acid (AA) substitutions with related physiochemical properties. Using the program AlphaFold (18, 19) PCPE and PCPE2 are predicted to have similar overall 3-dimensional structures, including a “linker region” connecting CUB2 and the NTR domain characterized as an intrinsically disordered region (IDR) (20). However, AlphaFold and MobiDB (21, 22) disagree on the IDR designation for PCPE2, suggesting there may be unique structural differences between the linker regions within PCPE and PCPE2. Since an excellent review of PCPE compared to PCPE2 has been recently published (23), the primary focus of this review will be to cover structural considerations of PCPE2. Additionally, this review will highlight reports from the current literature describing studies of PCPE2 expression and its association with clinically relevant disease states in humans and in animal models.

PCPE and PCPE 2 are both found in vertebrates, from mammals to fish, and possibly in more primitive members of phylum Chordata. Similarities and differences between PCPE and PCPE2 can be initially appreciated from a simple comparison of their domain AA composition compared to each other, shown in Table 1. In this Table, the percentage of homology between PCPE versus PCPE2 is shown for different species starting with humans. For instance, we see that the CUB2 domain is the most highly conserved between the two proteins (at ∼60% in humans to ∼68% in Coelacanth), while the NTR domain shows the least conservation (at 38% in humans to 54% in Coelacanth). Further insight into the structural conservation of the PCPE proteins, can be appreciated from an AA homology comparison of each PCPE protein’s own domain among different species. To do this the program Blast+ (16, 24) was used and the results are shown in Table 2. In this table, the first column compares the % identity or homology of PCPE or PCPE2 from human to Coelacanth, while the remaining columns show the % identity for each of the individual domains i.e., CUB1, CUB2, linker domain, and NTR domain. Because of the great potential of mouse models, both human and murine PCPE protein sequences were included, while other phylogenetic classes have only a single representative species. Overall, the analyses show a good match among sequences from mammals (human) to fishes (coelacanth). However, striking differences are seen immediately between the conservation of identity comparing PCPE and PCPE2. For example, Coelacanth PCPE2 exhibited ∼81% identity compared to humans, while the same comparison for PCPE was ∼51% conserved identity. Comparison of individual domains shows the same trend, in every instance a higher % identity for PCPE2 domains among species compared to PCPE. Interestingly, Table 2 suggests that there is little variation in the number of AA in most domains, however, the length of the IDR region in PCPE among species is quite variable and considerably different than in PCPE2 (17). The relative lack of AA conservation observed in the PCPE sequence among species is reflected in most of the individual domains, with the NTR domain showing the largest drop in conservation (45.8%) comparing coelacanth to humans while PCPE2’s NTR shows 75.6% identity when coelacanth is compared to the human sequence. However, the greatest change in %identity was found in the IDR region where PCPE % identity was ∼16% from humans to fish while PCPE2 was significantly greater at ∼64% identity for the same comparison. Overall, these data suggest a significant divergence in PCPE from 2’s sequence evolution and suggest that the linker domain may hold the key to understanding the unique functions of these two proteins.

**Table 1:** Percent Identity Comparing PCPE 1 versus 2

Species	DomainAAs %	CUB1 Domain %	CUB2 Domain %	Linker Domain %	NTR Domain %
Human	45.6	54.0	60.7	0	38.0
Mouse	44.8	56.7	65.5	0	40.5
Wombat	44.9	49.5	63.7	0	37.5
Platypus	44.8	52.7	65.2	0	38.4
Chickadee	47.7	58.9	63.4	0	36.9
Lizard	41.1	60.7	65.7	0	40.0
Frog	48.2	55.3	64.8	0	39.5
Coelacanth	61.0	67.3	67.8	0	54.2

**Fig. 1:**
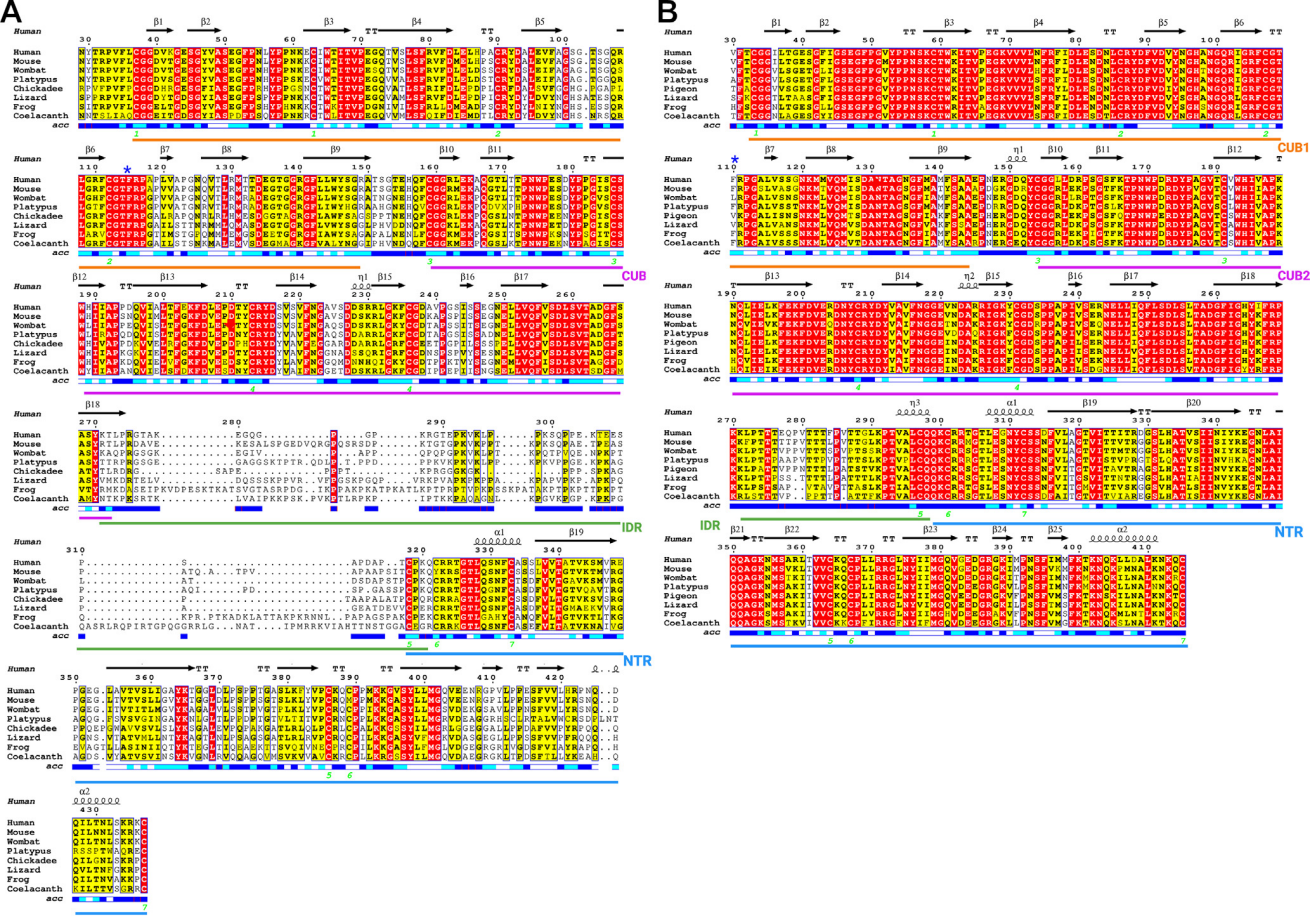
Comparison of PCPE1 and PCPE2 Amino Acid Sequences. Alignment of PCPE1 (A) and PCPE2 (B) from each of 8 species in 7 classes of vertebrates. Numbering at the top starts with amino acid 29 of human PCPE1 (Uniprot identifier-Q15113) and amino acid 30 of human PCPE2 (Uniprot identifier-Q9UKZ9). Secondary structure features for human PCPE1 or PCPE2 are listed at the very top, eg,→ β-strand, labeled sequentially as β-1 to β-n; e.eee, η is a 3_10_-helix, labeled from η-1 through η-n; e.eee, α is an a-helix labeled from α-1 to α-n; and strict β-turns as TT. Key: Red A strict identity; Yellow **A** similarity within a group; Yellow A moderate identity-chemical similarity; boxed sequences indicate similarity across groups, eg, (A) residues 33 through 38. Solvent accessibility (*acc*), blue = accessible, cyan = intermediate accessibility, and white, buried. CUB1 is identified by an orange line, CUB2 is identified by a purple line. The linker region is also designated as IDR, indicated by a green line, classified as an intrinsically disordered region predicted only in PCPE1. While in PCPE2 the CUB2 to NTR region is referred to as a linker region. The NTR is identified by a blue line. Blue asterisk indicates critical phenylalanine (F) for enhancing proteolysis in Pcpe1.

Percent identity comparing indicated domains which include amino acids (AA) 37–437 for PCPE1, and 33–415 for PCPE2. Numbering at the top starts with amino acid 29 of human PCPE1 (Uniprot identifier-Q15113) and amino acid 30 of human PCPE2 (Uniprot identifier-Q9UKZ9) illustrated in Fig. 1. The disordered region linking CUB2 and the NTR in PCPE1 and the linker region in PCPE2 have zero similarity.

**Table 2:** Percent Identity Comparing Pcpes Among Species

Species	PCPE1 All Domain AAs	PCPE2 All Domain AAs	PCPE1 CUB1 Domain%	PCPE2 CUB1 Domain%	PCPE1 CUB2 Domain%	PCPE2 CUB2 Domain%	PCPE1 Linker Region%	PCPE2 Linker Region%	PCPE1 Linker #AAs	PCPE2 Linker #AAs	PCPE1 NTR Domain%	PCPE2 NTR Domain%
Human	100.0	100.0	100.0	100.0	100.0	100.0	100.0	100.0	50	28	100.0	100.0
Mouse	85.6	89.1	95.7	92.9	89.7	92.2	31.8	85.7	68	28	83.3	86.6
Wombat	76.7	87.0	87.1	89.3	86.2	87.8	40.5	75.0	50	28	71.7	85.7
Platypus	67.2	86.0	81.9	92.9	78.4	90.4	31.8	71.4	62	28	50.8	79.8
Chickadee	59.2	83.9	62.9	85.7	70.7	89.6	34.4	71.4	39	28	53.3	79.0
Lizard	60.3	85.2	67.2	84.8	75.9	93.9	25.0	74.1	62	27	54.2	80.7
Frog	48.2	81.8	58.6	89.3	61.2	87.8	20.9	57.7	103	26	39.2	74.0
Coelacanth	51.1	81.5	53.4	80.4	69.0	83.5	15.9	64.0	92	25	45.8	75.6

Percent identity comparing indicated domains which include amino acids (AA) 37–437 for PCPE1, and 33–415 for PCPE2. Numbering at the top starts with amino acid 29 of human PCPE1 (Uniprot identifier-Q15113) and amino acid 30 of human PCPE2 (Uniprot identifier-Q9UKZ9) shown in Fig. 1. Numbering refers to Uniprot identifier sequence which includes the signal peptide corresponding to residues 1–25 for PCPE1 and 1–21 for PCPE2. Comparison among individual domains for PCPE1; CUB1 includes residues 37–149; CUB2, 159–273 while for PCPE2; CUB1 includes residues 33–144; CUB2 154–268. Linker #AAs refer to the number of residues in that region and correspond to the designation of a disordered region for PCPE1 including residues 271–321, while in PCPE2 the region which connects CUB2 with the NTR domain is referred to as the “Linker” region and includes residues 271–299. For the NTR region the residues include for PCPE1 318–437 and for PCPE2 297–415. The percent identity was calculated using the program Clustal Omega (Madeira F, *et al.*, (2022) *Nucleic Acids Res* 50:276-279).

A comprehensive visualization of AA similarities and differences among 8 species, is also shown in Fig. 1 using alignment programs Clustal Omega (25, 26), T-Coffee (27), and ESPript 3.0 (17). Figure 1A shows the primary sequence consensus alignment among species for PCPE, and in Fig. 1B the same for PCPE2, with the order of species listed from top to bottom and the same as in Table 1, Table 2. Structural elements, eg, ⍺-helical regions, turns, and β-sheets, are from AlphaFold analysis of human PCPE and PCPE2 with AlphaFold identifiers AF-Q15113-F1-v4 and AF-Q9UKZ9-F1-v3, respectively. Residues are highlighted red if they are identical, highlighted yellow if they are similar (eg hydrophilic or hydrophobic), and have no highlight if dissimilar. As was discussed in Table 2, casual inspection of panels A and B in Fig. 1 shows a much higher percentage identified in PCPE2 compared to PCPE. Also apparent from Fig. 1 is the large difference in the number of AA in the IDR or linker region between PCPE and 2, as well as the lack of conservation among species within the PCPE IDR relative to PCPE2. Also of note, are the key elements of the CUB domains which are highly conserved across species with numerous cysteines that are believed to participate in disulfide bonds locking these domains and forming distinct conformations (28). Interestingly, the CUBs of PCPE and 2 both belong to a unique group of calcium-binding CUBs (cbCUB) (9), with X-ray analyses of proteins possessing cbCUB domains suggesting that up to three acidic AAs associated with Ca^+2^, as this stabilizes interaction within the CUB domain (9).

Although PCPE has been studied more extensively than PCPE2, the similarity in their domain organization suggests, but does not definitively prove, that structural results reported for PCPE may also apply to PCPE2. Amino acid alignments of CUB domains in PCPEs and other proteins indicate that CUB domains are highly conserved (9, 28). Extensive X-ray analysis of the (procollagen C-propeptide)-PCPE complexes suggests that Ca^2^⁺ ions and acidic amino acids, likely aspartic acids (D), within PCPE, are crucial for procollagen binding. For example, in one report, replacing two highly conserved Ds in the CUB1 domain with alanine (A) reduced the enhancement of procollagen cleavage (27), as did replacing one phenylalanine (F) at position 90 in CUB1 with alanine (A). This showed that F90 was essential for PCPE binding to procollagen and enhancing proteolysis (28), F90 is marked by a blue asterisk in Fig. 1 Panels A and B. From inspection of Fig. 1, it should be noted that F90 in PCPE is highly conserved among species, while in PCPE2 very little conservation is noted.

Visual inspection of AlphaFold models AF-Q9UKZ9-F1-v3 (PCPE2) compared to AF-Q15113-F1-v4 (PCPE) suggests a similar organization for PCPE2. To visualize this, an X-ray analysis of the PCPE CUB1-CUB2 complex with the C-propeptide trimer of procollagen III (CPIII) was conducted. The analysis revealed that PCPE’s CUBs interact with CPIII, with the interaction stabilized by highly conserved acidic residues and a tyrosine residue in PCPE, likely involved in Ca^2^⁺ coordination and suggesting, that PCPE and CPIII form a 1:1 complex (29, 30). Based on the alignments of CUB1-CUB2 in PCPE and 2 from various species by us and others, it is anticipated that PCPE and 2 will have similar 3-dimensional conformations within their CUB and NTR domains (15, 28). However, despite multiple structural similarities between PCPEs 1 and 2, accumulating evidence suggests that PCPE and 2 possess distinct biochemical functions.

Reported post-translational modifications of PCPE2 include phosphorylation and glycosylation under specific stimuli or conditions. According to one report, PCPE2 has up to eight phosphorylation sites (31) including serine phosphorylation in the CUB1 region when isolated from a human Jurkat T cell leukemia line (32) and in the NTR region from isolates of Chang liver cells (33). Additionally, tyrosine and serine phosphorylation has been reported in the CUB2-linker region of PCPE2 isolated from human ovarian and breast cancer xenograft tissue (34). While human PCPE2 has potential N-glycosylation sites (35) human PCPE2 did not exhibit a SDS-PAGE mobility shift after treatment with Peptide N-Glycosidase F (PNGase F), suggesting little to no N-glycosylation. In contrast, PCPE showed mobility changes after PNGase F treatment (35). While following treatment of PCPE2 with a mixture of sialidase A and endo-O-glycosidase, SDS-PAGE mobility changes indicated that PCPE2 has O-glycosylation sites with Galβ-(1–3)-GalNAc O-linked cores (34). Recently, Yang *et al.*, using mass spectrometric methods, identified an O-linked Hex-HexNAc at T270 of PCPE2 (36, 37). Overall, these analyses suggest that PCPE and PCPE2 have distinct patterns of O- and N-linked glycosylation, which may influence their biological functions.

## PCPE and 2 in procollagen processing

Early studies have demonstrated that PCPE and PCPE2, which are located within the ECM, facilitate the enzymatic processing of C-propeptides from procollagens, thereby promoting the synthesis of major fibrillar collagens (8, 14, 38, 39, 40, 41, 42, 43). One enzyme involved in this processing is the bone morphogenetic protein 1 (BMP-1) a splice variant of mammalian Tolloid (mTLD), also known as type I procollagen C-proteinase (44, 45, 46). It should be noted that both mTLD and mTLL1 also have procollagen C-proteinase activity and are also regulated by PCPE and PCPE2 (15). Initial findings indicated that PCPE could stimulate C-propeptide processing by up to 20-fold (1), whereas it did not enhance the processing of other BMP-1 substrates (15, 38). More recent studies have revealed that PCPE2 modulates procollagen C-propeptide cleavage by BMP-1 in a concentration-dependent manner, moderately increasing cleavage at low concentrations, while significantly inhibiting cleavage at higher concentrations (15). Additionally, studies also show that PCPE2 inhibits BMP-1 cleavage of thrombospondin-1 (THBS1) (47), beta glycan (TGFBR3), chordin (CHRD), and the low-density lipoprotein receptor (LDLr) (15). PCPE2 also enhances the removal of a propeptide (48) from proapolipoprotein A-I (proapoA-I), resulting in the production of mature apoA-I, the major protein component of plasma high-density lipoprotein (HDL) (49). These findings suggest that both PCPE and PCPE2 bind to procollagens I and II thereby modulating BMP-1 proteolysis (35). It has also been reported that PCPEs displace mTLD from procollagens I and II (35), supporting the notion that PCPE binds to the trimeric procollagen substrate, alters its conformation, and makes individual chains more accessible for cleavage (14). Subsequent research showed that the interaction between BMP-1 and the PCPE-substrate complex induces a conformational change in BMP-1 (30). Low-resolution structures of BMP-1 and mTLD reveal that the non-catalytic CUB and EGF domains of PCPE partially obscure the active site of these proteases (50). This mechanism contrasts sharply with other ECM metalloproteinases, such as matrix metalloproteinases (MMPs), whose activities are regulated by endogenous inhibitors rather than enhancer proteins. As mentioned above, recent reports indicate that PCPE2 can inhibit BMP-1 activity, opposite to the enhancing effect of PCPE on activity (15). Thus, despite the distinct tissue distribution and glycosylation patterns of PCPE and 2, there remains limited research on whether these glycoproteins have additional divergent functions, raising the question of whether in vitro studies clearly reflect in vivo physiology.

The contribution of PCPE and PCPE2 in corneal scarring-repair has been extensively studied using fibroblasts which are reported to express vastly more PCPE than PCPE2 (35). Studies show that in both mouse and human corneas using the corneal excision method, BMP1 and mTLD mRNA, and protein expression, was increased in response to wounding as was PCPE mRNA expression (51). In contrast, PCPE2 mRNA abundance decreased significantly below the level reported in unwounded tissue (51). The authors conclude that PCPE has a major role in collagen fibrillogenesis during wound healing, while PCPE2 may only be involved in the early stages of this process. Corneal wound healing after alkali burn injury in keratocytes from *Pcolce* knockout mice (*Pcolce*-KO) showed a reduction in procollagen 1 processing, while neo-angiogenesis was enhanced (52); however, this difference did not correspond to discernible differences in corneal opacity or collagen content and suggest that PCPE may play an important anti-angiogenic factor in re-epithelialization following injury in cornea. In contrast to Pcpe, mouse cornea had nearly undetectable levels of Pcpe2 protein as compared to levels found in murine heart tissue (52). In a second study in the global *Pcolce*-KO mice model the loss of *Pcolce* modestly reduced retinal vascular density compared to control mice and inhibited both cell proliferation and tube formation of retinal microvascular endothelial cells (53). Collectively, these in vivo studies support the notion that Pcpe, but not Pcpe2, enhances procollagen 1 processing for retinal angiogenesis.

Studies found no differences in collagen fibrils from the skin of *Pcolce2*-KO mice compared to wild-type mice, which is consistent with the idea that Pcpe2 does not stimulate the activity of BMP-1(15). Rather *PCOLCE2* appears to be a potent inhibitor of BMP-1 mediated processing of procollagen I maturation (15). When subjected to cardiac pressure overload induced by transverse aortic constriction hearts from global *Pcolce2*-KO mice, the authors found lower collagen content, less muscle stiffness, and greater survival compared to control mice (54) despite collagen levels in unstressed hearts from *Pcolce2*-KO and wild-type mice being identical. Hearts from stressed *Pcolce2*-KO mice also showed normal *Pcpe* expression indicating that Pcpe does not increase to compensate for the loss of Pcpe2, with the authors concluding that the role of Pcpe2 was only unmasked in circumstances of cardiac stress. In other studies, global *Pcolce*-KO mice showed no major difference in cardiac collagen levels or cardiac function under basal conditions as determined by echocardiography (55) and no differences in survival, clinical chemistry, or organ histology when *Pcolce*-KO mice were compared with wild-type or control mice. In zebrafish, midkine-a (MDKA), a small cytokine upregulated during inflammation/injury in several tissues, including heart, was studied using *midkine-a* knockout (*mdka*-KO) zebrafish hearts (56). Following injury, *mdka*-KOs had defects in regeneration potential compared to controls. Transcriptional analysis of the injured heart tissue revealed a significant increase in *pcolce2b* mRNA expression along with mRNAs from tumor growth factor β type III receptor (*tgfbr3*), collagen genes (56), basic helix-loop-helix transcription factor (*twist1*), and caveolin-1 (*cav1*) (57). Therefore, collectively these studies and others suggest that PCPE2, like PCPE, plays a role in procollagen processing, but that this role may take place in specialized fibroblasts which may set it apart from PCPE (58, 59).

## Insights from single-cell RNA (scRNA) sequencing: fibroblasts and precursor cells

The widespread use of scRNA seq analyses of mouse and human tissues has yielded new insights into the expression and function of PCPE2. Recent research has identified several novel fibroblast types within human prostate tissues, each with unique distributions and microenvironmental interactions (60). Among these, *PCOLCE2* was found to be highly expressed in a subtype of fibroblasts localized to the interstitial spaces, termed interstitial fibroblasts. These cells were present throughout all prostate anatomical zones except near the ejaculatory ducts, where they expressed complement component 7 (*C7*), cholecystokinin (*CCK*), and gelsolin (*GSN*) mRNA (60).

In another study, scRNA-seq analysis of human skin fibroblasts revealed a population expressing secreted frizzled-related protein 2 (*SFRP2+*), which was further divided into two subpopulations. The first, known as Wingless and Int-1 (*WNT*) inhibitory factor 1 (*WIF1+*) cells, expressed cartilage oligomeric matrix protein (*COMP*), a negative regulator of WNT signaling 2 (*NKD2*), and exhibited high levels of type I collagen. The second subtype, *PCOLCE2+* cells, expressed *PCOLCE2*, complement decay-accelerating factor (*CD55*), and follistatin-like 3 (*FSTL3*), and were located adjacent to the *SFRP4+* cell population. This proximity suggests that *SFRP4+* cells might be progenitors of *PCOLCE2+* and/or *SFRP2+* cells (61). Given that *SFRP2* and *SFRP4* are known modulators of the WNT signaling pathway, it is possible that PCPE2 plays a role in these processes. Consistent with this, scRNA-seq of cryopreserved human skin biopsy tissues identified a distinct myofibroblast cluster marked by genes including *WIF1*, *NKD2*, *PCOLCE2*, secretory leukocyte peptidase inhibitor (*SLPI*), *CD55*, actin alpha 2 (*ACTA2*), and *WNT* Family Member 2 (*WNT2*) Collectively, these findings suggest that *PCOLCE2* is expressed in specialized fibroblast cells that significantly contribute to fibrosis onset and cell fate determination, possibly working through the Wnt signaling pathway.

Research involving transforming growth factor β receptor 2 (*Tgfbr2*) knockout mice revealed that while tendon development proceeded normally despite the loss of this receptor, tendon cells began to lose their differentiation markers shortly after birth, reverting to a stem/progenitor state (62). Notably, scRNA-seq showed an 11.5-fold decrease in *Pcolce2* mRNA expression, aligning with other markers suggesting an association with a loss-of-cell fate phenotype (62). In another study, unsupervised clustering analysis of cartilage transcriptomes linked *PCOLCE2* mRNA regulation to an osteoarthritis subtype known as glycosaminoglycan metabolic disorder (63). This disorder is associated with glycosaminoglycan and, chondroitin sulfate metabolic processes and is linked to ECM organization and cell adhesion, involving TGFb1 and metalloproteinase expression. In another study of osteoarthritic cartilage, the authors report and confirm the expression of two genes not previously identified in cartilage biology, namely *PCOLCE2*, and N-acetylgalactosaminyltransferase 15 (*GALNT15*) (64). It is believed that since PCPE2 plays a role in collagen processing, and cartilage is composed of collagen type II, these genes may provide biomarkers for these processes. In other studies of Dupuytren’s contracture, a fibroproliferative disease, *PCOLCE2* mRNA and PCPE2 expression were observed to be twice as high in the cord form of the disease compared to the nodular form. In contrast, *PCOLCE* mRNA levels remained relatively unchanged across both forms (65). Together these studies again suggest that *PCOLCE2* is associated with the TGFb pathway and cell fate determination.

Investigations into human skeletal muscle identified PCPE2, rather than PCPE, as a marker for fibro-adipogenic progenitor cells (FAPs) during fatty infiltration (66). The *CD55+* FAP population was characterized by differential expression of tenascin XB protein (*TNXB*), microfibril-associated protein 5 (*MFAP5*), *PCOLCE2*, Fibrillin 1 (*FBN1)*, *CD55*, and proteoglycan 4 (*PRG4*), all of which have been associated with synovial cell and chondrocyte expression. Skeletal muscle FAPs serve as precursors to specialized cells, including activated fibroblasts, adipocytes, and osteoprogenitor cells (67). As summarized by Negroni *et al.*, (68) *PCOLCE2* shows a strong association with adult human muscle in three other unbiased scRNA-seq studies of FAP populations involved in muscle homeostasis (69, 70, 71). Additionally, using the Genotype-Tissue Expression database, a whole-genome expression analysis revealed significant downregulation of *PCOLCE2* mRNA in T2D patients compared to age-, gender-, and race-matched nondiabetic controls across various tissues (72, 73). These findings suggest potential signaling network defects in human skin that could serve as predictive markers for evaluating treatments for T2D-related skin disorders (72).

In mucopolysaccharidoses (MPS), a group of lysosomal storage diseases associated with neurodegeneration*, PCOLCE2* along with three other genes, was found to be up-regulated across all MPS types and subtypes (74). Analysis of single-cell transcriptome data from both intact and injured mouse peripheral nerves revealed that *Pcolce2*, together with *Sfrp2*, dermatopontin *(Dpt),* ADAM metallopeptidase with thrombospondin type 1 motif 5 (*Adamts5)*, peptidase inhibitor 16 *(Pi16)*, *Sfrp4*, paired-related homeobox 1 (*Prrx1*), cartilage oligomeric matrix protein (*Comp)*, and lymphocyte antigen 6 family member C1 (*Ly6c1)*, served as marker genes for identifying epineurial fibroblasts involved in nerve regeneration and wound healing (75). Moreover, *PCOLCE2, LY6C1*, and dipeptidyl peptidase 4 (*DPP4*) emerged as significantly regulated biomarkers for epineural mesenchymal cells, with scRNA-seq identifying *PCOLCE2* as a key biomarker for cell clusters undergoing peripheral nerve cell regeneration (76, 77). These findings collectively suggest that *PCOLCE2* is expressed by specialized fibroblasts in various tissues and plays a crucial role in cellular repair and development.

## PCPE2 and lipid metabolism

The connection between lipid metabolism and PCPE2 was initially suggested when BMP-1 was identified as the protease responsible for processing secreted pro-apoA-I into its mature form, apoA-I (48). In 2009, Zhu *et al.* (49) showed that PCPE2 bound to BMP-1 and pro-apoA-I accelerated proteolytic removal of the pro-peptide on apoA-I (78). Later studies using *Pcolce2-KO* mice showed nearly a twofold increase in plasma concentrations of enlarged HDL particles, suggesting that HDL elevation resulted from a decreased cholesterol efflux to pro-apoA-I (79, 80). This prompted further investigation into whether high plasma concentrations of enlarged HDL would be protective in *Pcolce2-KO* mice from atherosclerosis (81), since data from the Framingham Heart Study indicate that higher plasma HDL concentrations are associated with protection from atherosclerosis in humans (82). To assess the level of protection, global *Pcolce2-KO* mice were crossed with low-density lipoprotein receptor-KO mice (*Ldlr-KO*) to produce *Pcolce2*, *Ldlr-*double (DKO) mice. When these mice were fed a Western diet, they exhibited a 46% increase in lipid deposition and CD68 macrophage staining in the aortic root compared to *Ldlr-KO* control mice on the same diet. This finding suggested that the elevated plasma HDL concentration in *Pcolce2-KO* mice was not athero-protective. Subsequent crossover experiments investigated whether the increased atherosclerosis was due to dysfunctional HDL from *Pcolce2, Ldlr*-DKO mice or impaired HDL metabolism. HDL isolated from both *Pcolce2, Ldlr-*DKO and *Ldlr-KO* mice were catabolized at the same rate in *Ldlr-KO* mice but were catabolized more slowly in *Pcolce2, Ldlr*-DKO mice. This indicated that the slower HDL catabolism in *Pcolce2, Ldlr-*DKO mice contributed to higher plasma HDL levels and increased atherosclerosis, rather than indicating HDL dysfunction in the absence of Pcpe2 (81).

Follow up studies that measured the uptake of fluorescently labeled HDL by the scavenger receptor class B type 1 (Srb1, gene name *Scarb1*) expressed in differentiated stromal vascular adipocytes from, e.g., *Pcolce2*, *Ldlr*-KO cells versus *Ldlr*-KO cells, showed that preincubation of *Pcolce2*, *Ldlr*-KO stromal vascular adipocytes with exogenous PCPE2 restored Srb1–mediated HDL-C uptake (83). The *Pcolce2* knockout yielded a phenotype like that reported for *Scarb1-KO* mice, which also exhibit increased atherosclerosis despite elevated plasma HDL levels (84). Therefore, without functional Srb1, HDL particles continuously recirculated in plasma becoming more cholesterol enriched and thus contributing to the process of atherosclerosis rather than protecting against it. Both human and mouse studies have shown that *PCOLCE2* mRNA levels in visceral and subcutaneous adipose tissue are positively correlated with absolute fat mass (83). Because HDL uptake is crucial for maintaining plasma to adipose lipid balance, it seems likely that *Pcolce2, Ldlr*-DKO mice overexpresses liver Srb1 in order to enhance HDL uptake (81, 83). In addition to elevated plasma HDL cholesterol levels, both *Scarb1*-KO and *Pcolce2, Ldlr*-DKO mice exhibited higher plasma triglyceride concentrations compared to normal controls (85).

Several studies using the Framingham Heart Offspring Cohort have applied genome-wide association studies (GWAS) to explore correlations between red cell arachidonic acid levels and single nucleotide polymorphisms (SNPs) in *PCOLCE2* (86). Follow-up studies identified *PCOLCE2*, along with 12 other loci, as significantly associated with plasma fatty acid levels (87). In studies specifically addressing how adipose tissue responds to transitioning from fasting to the fed state, researchers found *Pcolce2* levels were elevated in response to TGFb treatment while the levels were suppressed in adipose tissue from mice lacking mothers against decapentaplegic-3 (*Smad3-KO*) mice. Furthermore, these studies also showed that as ECM-related genes increased in response to the fed state, as did a number of lipogenic genes including *Ldlr*, *Fasn*, and *Glut4* (59), suggesting a coordinated mechanism of adipose remodeling during plasma lipid uptake and storage. When control mice were treated with anti-TGFβR1 antibodies a significant reduction in visceral and subcutaneous (VAT and SAT) fat mass was observed along with a reduction in the abundance of *Pcolce2* mRNA. These observations are consistent with the significant reduction in VAT and SAT mass observed in *Pcolce2, Ldlr*-DKO mice (83).

In chronic hyperglycemia, associated with type 2 diabetes (T2D), insufficient insulin secretion from pancreatic β-cells usually occurs. Recently, Bacos *et al.* (88) performed sc-RNA-seq of pancreatic islet cells from over 300 T2D patients and controls and found 395 DEGs. Of these, bar subclass of homeobox transcription factors (*BARX1*), neurofilament light chain (*NEFL*), paired box 5 (*PAX5*) and *PCOLCE2*, were selected for functional follow-up studies (88). In virally transduced INS1β cells, elevated expression of *NEFL* and *PCOLCE2* was linked to significantly impaired glucose-stimulated insulin secretion at both basal and stimulatory glucose levels, as well as increased insulin content (88). Selected DEGs that exhibited higher islet expression in T2D, *BARX1*, extracellular leucine-rich repeat and fibronectin type III domain containing 1 (*ELFN1*), Fas apoptotic inhibitory molecule 2 *(FAIM2*), *NEFL*, *PAX5*, *PCOLCE2* and *SFRP1* were overexpressed by plasmid transfection in INS1β cells. Under basal or glucose-stimulated conditions, cells overexpressing *Pcolce2* showed impaired insulin secretion. The authors also examined SNPs associated with the identified DEGs T2D and metabolic traits and found 148 SNPs associated with islet expression of several genes including *PCOLCE2*. Six eQTL SNPs associated with islet expression of *PCOLCE2* (rs6794287) have been linked to triglyceride or LDL cholesterol levels. From these studies the authors conclude that *PCOLCE2* expression is upregulated in islets of T2D patients and impairs insulin secretion through as yet an unknown mechanism (88). Collectively, these studies strongly suggest that *PCOLCE2 and not PCOLCE* expression is associated with progenitor cell identity and/or their ability to differentiate.

## PCPE2 and inflammation

The knockdown of *PCOLCE2* in tonsil-derived mesenchymal stem cells cocultured with differentiated human leukemia 60 (dHL-60) cells, hindered the enhancement of reactive oxygen species (ROS) production by these cells (89). Neutrophil-generated ROS serves as a frontline defense against invading pathogens, suggesting PCPE2 holds promise for modulating these defense mechanisms. Addition of recombinant PCPE2 directly augmented ROS production, correlating with increased NADPH oxidase (*NOX*) 3, 4, and 5 mRNA levels in HL-60 cells. Overall, these findings imply that PCPE2 may enhance frontline defense by boosting ROS generation in neutrophils. RNA sequencing of neutrophils from children with untreated idiopathic arthritis, cystic fibrosis, or controls identified *PCOLCE2* as one of the top 10 differentially expressed genes, suggesting specificity in neutrophil transcriptional responses across disease states (90). Transcriptomic profiling identified a module of 26 genes, including *PCOLCE2*, predictive of glycoprotein acetylation, inflammation, activated neutrophils, and infection risk (91). In the context of sepsis, which involves complex causative factors and a broad inflammatory response similar to severe inflammatory response syndrome of non-infectious origin (SIRSNO), distinguishing between these conditions is challenging. A meta-analysis using artificial neural networks on SIRS/sepsis and other infection datasets identified eight core hub genes, including PCOLCE2, associated with SIRS/sepsis immunopathology (92). Despite strong interactions among these genes, current methods do not sufficiently differentiate between SIRS and sepsis based on gene expression alone. In a bioinformatic study that examined two publicly accessible gene expression profiles from the Gene Expression Omnibus database, authors focused on identifying genes exhibiting differential expression between severe and non-severe influenza patients (93). Ten of these, including *PCOLCE2*, *HLA DPA1*, and *MPO* were extensively studied. Functional studies showed that, unlike *HLA DPA1*, *PCOLCE2* levels were significantly higher in the severe influenza group compared to the non-severe influenza group. These studies demonstrated from ROC curves that only *PCOLCE2* and *HLA DPA1* had area under the curve above 0.7 but focused on HLA DPA1 since enrichment analysis indicated that this gene was associated with adaptive immune responses and inflammation-related pathways. As noted in other studies, the role *PCOLCE2* plays in severe influenza immune response remains unknown.

Intestinal inflammation elicits a dynamic fibroblast response, and mesenchymal cell responses play a pivotal role in determining whether pathological fibrosis ensues, or healing and resolution are achieved. To better understand the cellular and molecular circuitries operating during intestinal inflammation, ECM remodeling, and wound healing, a murine model of colonic inflammation based on oral administration of multiple cycles of low-dose dextran sulfate sodium (DSS) was used to induce epithelial injury and ECM deposition (94). Stromal cell heterogeneity in response to chronic inflammation in the colon using the DSS model was investigated by sc-RNA sequencing of colon-derived stromal cells. *PCOLCE2* was found to serve as a marker gene for Fibroblast 3 cells (94) which do not express genes related to regulation of growth factors necessary for crypt architecture. In contrast to Fibroblast 1 and 2 subsets, which maintain colon crypt architecture through opposing WNT and bone morphogenic protein (BMP) gradients within the transforming growth factor beta (TGFβ) superfamily. Fibroblast 1 and 2 subsets express atypical chemokine receptor 4 (*ACKR4*), acting as a chemokine sink to regulate gradients. The data presented suggest that Fibroblast 3 cells are mesenchymal cells that remodel the ECM in the intestine. These cells have also been referred to as crypt bottom fibroblast 2 (CBF2) or interstitial stromal cells that express peptidase inhibitor 16 (*Pi16*), cluster of differentiation 81 (*CD81*), and are platelet-derived growth factor receptor alpha 1 *(Pdgrfa1)* low. Importantly, these *Pi16*-expressing interstitial fibroblasts are a universal fibroblast subset found in all tissues. Specifically, Fibroblast 3 cells, coexpressing *Pcolce2*, *CD55*, *Pi16*, and complement component 3 (*C3)*, were localized within the muscularis mucosa. Taken together these studies indicate that a subset of matrix-depositing *Sfrp2+Dpp4+* fibroblast share expression of key lineage markers, *Pcolce2+* and *CD55+*, suggesting they participate in a concerted fibroblast differentiation program giving rise to myofibroblasts that promote wound healing in diverse tissues including the intestinal mucosa (94).

Other studies have highlighted the crucial role of the transcription factor zinc finger E-box binding homeobox 2 (*ZEB2*) is essential for maintaining tissue-specific identities of macrophages. Deletion of *ZEB2* results in the loss of various macrophage populations across tissues, including liver receptor α (*LXRA*, *NR1H3*), crucial for Kupffer cell identity (95). Intriguingly, *PCOLCE2* expression is significantly downregulated in splenic macrophages following ZEB2 deletion. In one study, transcriptome profiling and bioinformatic analysis were used to define the unique expression of ECM-associated genes in cultured macrophages. Identification of genes most regulated in the core matrisome included *PCOLCE2* expression (96). Furthermore, interferon-gamma treatment induced substantial downregulation of PCPE2 in the core matrisome of monocyte-derived macrophages. These findings reinforce the concept that macrophages are critical regulators of inflammation and tissue remodeling, providing structural integrity through the expression of core matrisome genes (96).

Using a mouse model of chronic pancreatitis with microarray analyses investigators found a two-fold upregulation of *Pcolce2* mRNA from pancreatic tissue comparing two strains of mice, namely the C57BL/6J and B6N strains. Other DEGs included matrix metallopeptidase 7 *(mmp7*), inter-alpha-trypsin inhibitor heavy chain 4 *(itih4*), wd repeat and FYVE domain containing 1 (*wdfy1*), neurofascin (*nfasc*), interferon-induced protein with tetratricopeptide repeats 1 (*ifit1*), placenta associated 9 (*plac9*) and vitronectin (*vtn)* (97). The data showed that a striking difference was observed during induction of chronic cerulein-induction, with more severe pancreatic atrophy, inflammatory cell infiltration, and fibrosis in the B6J versus the B6N. Overall, this comparison identified a more severe phenotype which included *Pcolce2* expression and linked PCPE2 expression with genes associated with fibrosis and cell adhesion.

## PCPE2 and cancer

Cancer is a heterogeneous disease that poses significant challenges for personalized treatment at the individual patient level. Both bulk and sc-RNA-seq technologies have been employed to study the transcriptional profiles of tumors at the gene expression level. Recent studies highlight *PCOLCE2* expression as a valuable biomarker with prognostic and predictive significance. Additionally, hub genes, that have been shown to link various types of cancers through networks of differentially expressed genes, can be analyzed to determine their clinical utility in predicting outcomes.

Among the studies reviewed, *PCOLCE2*, rather than *PCOLCE*, has emerged as a highly significant prognostic biomarker, particularly in colon and colorectal cancers (98, 99, 100, 101, 102, 103, 104, 105, 106, 107). Colorectal cancer, the most commonly diagnosed gastrointestinal malignancy, ranks third in morbidity and second in mortality, with an estimated 3.2 million new cases projected for 2024. Research has identified *PCOLCE2* as a novel gene signature for high-risk groups in age-stratified analyses of colorectal cancer patients (99). Additionally, *PCOLCE2* was among nine key prognostic genes reported in colon adenocarcinoma (100) and was included in a prognostic gene group of nine in integrated bioinformatics analyses of colorectal cancer (101). Furthermore, a ferroptosis-related gene signature associated with invasion and metastasis in colon adenocarcinoma identified *PCOLCE2* as one of four key prognostic genes (98). Exome sequencing in a family with gastric and rectal cancer revealed twelve genes, including *PCOLCE2*, with non-synonymous single nucleotide variants shared among affected members (108). Collectively, these studies implicate *PCOLCE2* expression in colorectal adenocarcinoma and suggest that its involvement in ECM receptor interactions and the Wnt signaling pathway may contribute to tumor growth and metastasis.

In other cancers, such as ovarian cancer, PCPE2 expression in tumor-associated macrophages was found to contribute to cancer progression along with enhanced ECM reorganization and elevated ascites levels of interleukins 6 and 10 (IL-6 and IL-10). Combined, these factors may link *PCOLCE2* expression to the aggressiveness of ovarian cancer and immune suppression (109, 110) regulated in part by TGFβ1 (111). PCPE2 upregulation has been associated with metastasis in human ovarian tissues after chemotherapy (112), while similar associations were found with porcine ovarian granulosa cells that placed *PCOLCE2* as one of the ten highest DEG identified (113). Thus, these observations link up-regulation of the *PCOLCE2* expression with active remodeling of ECM composition in granulosa cells. Ongoing in vitro studies have also demonstrated increased expression of collagens (COL1A2, COL3A1, COL5A2, COL12A1, COL15A1, and COL6A3) in the theca interna of the bovine ovarian follicle, leading to their consideration as cellular markers of this structure Studies of human gynecological cancers showed *PCOLCE2* as a gene in cancer progression (114). While in a study investigating nasopharyngeal carcinoma 4 hub genes were identified with one being *PCOLCE2* (115). In gastric cancer, five epithelial-mesenchymal transition (EMT)-related genes were identified by single-sample gene set enrichment analysis and *PCOLCE2* was one of those genes that strongly correlated with patterns of tumor immune microenvironment (116). This finding was validated in a second study, where *PCOLCE2* was among four signature genes associated with progression and prognosis in gastric cancers (117).

In three independent reports on bladder cancer biomarkers, *PCOLCE2* was identified as one of a handful of signature genes that predict prognostic, and immunotherapy benefit (118, 119, 120). In thyroid cancer studies, *PCOLCE2* was found to be one of four tumor microenvironment-related genes significantly affecting prognosis (121). Furthermore, *PCOLCE2* emerged as a strong predictor in a four-gene signature for papillary thyroid carcinoma (122). In liver tissue from hepatectomized cholangiocarcinoma-associated fibroblasts, *PCOLCE2* was among several genes overexpressed compared to non-tumorigenic liver fibroblasts (123). A six-mRNA prognostic model for head and neck squamous cell carcinoma highlighted *PCOLCE2* as highly predictive (124).

Research on energy restriction in animal models shows it can reduce carcinogenesis rates. Although human studies are less extensive, the anticipated downregulation of metabolic genes, including *PCOLCE2*, associated with energy restriction was inconsistent between intermittent and continuous regimens (125). This inconsistency may influence breast cancer indicators in women. Protein-protein interaction network analysis identified nine key genes, including procollagen-α1(XI) (*COL11A1*), matrix metallopeptidase 11 (*MMP11*), and procollagen-α1(X) (*COL10A1*), which were highly expressed. *PCOLCE2*, along with ADAM metallopeptidase with thrombospondin type 1 motif 5 *(ADAMTS5)*, laminin subunit alpha 2 (*LAMA2*), transmembrane O-mannosyltransferase targeting cadherins 1 (*TMTC1*), tissue inhibitor of metalloproteinase 4 (*TIMP4*), and R-spondin-3 (*RSPO3*), showed reduced expression levels. A similar expression profile for the first seven genes was validated in MCF-7 and MDA-MB-231 human breast cancer cell lines (126).

Recent reports suggest PCPE2 may promote the uptake of anthocyanins that function in an antiatherogenic (127) and/or anticancer capacity (128). Additionally, a study on regional localization in the pituitary gland identified *PCOLCE2*, along with pituitary-specific positive transcription factor 1 (*POU1F1*), pro-opiomelanocortin (*POMC*), and neuronal pentraxin-2 (*NPTX2)*, as pituitary-enriched proteins localized primarily in the anterior lobe compared to the posterior lobe (129). This set of proteins offers potential insights into the biochemistry of functional and nonfunctional pituitary adenomas (129) and parathyroid hyperplasia (130). Single-cell RNA sequencing studies have shown that pancreatic islet cells from type 2 diabetes patients exhibit high levels of *PCOLCE2* mRNA along with several other genes (88). Employing KO murine models, expression of *Pcolce2* was associated with impaired glucose-stimulated insulin secretion (88). In pancreatic ductal adenocarcinoma research, PCPE2 protein levels were found to be lower than those of PCPE. Further analysis suggested that PCPE enhances BMP-1 suppression of metastasis, supporting the hypothesis that BMP-1 suppresses metastasis through procollagen cleavage (131).

A common feature across many of these cited reports is the involvement of the TGF-β pathway, which is frequently implicated in various tumors. This pathway has been extensively studied and is known to inhibit cell proliferation during the early stages of tumorigenesis while promoting epithelial-mesenchymal transition in advanced cancer, acting as a double-edged sword. Given the documented connection between *PCOLCE2,* TGF-β, and BMP-1 activity (132, 133, 134, 135) upregulation of PCPE2 may significantly influence cancer progression through modulation of this pathway (136, 137, 138, 139, 140, 141, 142). The global ECM proteome, or "matrisome," includes core ECM proteins such as collagens, proteoglycans, and ECM glycoproteins like *PCOLCE2*. In cancer research, hub genes are differentially expressed genes that connect various cancer types and influence networks of co-expressed genes, which may with time provide a means of predicting outcomes and guiding clinical applications (143).

## Conclusions and speculation

*PCOLCE2* exhibits high sequence similarity to *PCOLCE*, particularly in the CUB1-CUB2 regions, indicating that *PCOLCE2*, like *PCOLCE*, is involved in ECM protein interactions. PCPE2 binds to the C-propeptide trimer of procollagen III, suggesting that it influences collagen deposition and organization in a manner like PCPE. Recent studies have shown that PCPE2 modulates the proteolysis of specific substrates by inhibiting BMP-1 activity, which is crucial for processing ECM proteins and other substrates involved in tissue remodeling, wound healing, and repair. Additionally, PCPE2 regulates lipoprotein uptake through the lipid transporter, SR-B1 and plays a significant role in adipose tissue remodeling, implicating its involvement in lipid metabolism and adipose tissue homeostasis. This involvement suggests potential impact on metabolic disorders such as obesity and dyslipidemia. Recently, sc-RNA sequencing studies have highlighted *PCOLCE2’s* role in various physiological and pathological processes, including inflammation and cancer. While *PCOLCE2* participates in cell commitment pathways, its specific mechanistic roles and cellular functions remain to be fully elucidated. Therefore, in conclusion, *PCOLCE2* likely operates at the intersection of ECM biology, proteolysis, lipid metabolism, and cell signaling, exerting regulatory effects across diverse physiological and pathological processes. Although it shares similarities with *PCOLCE, PCOLCE2* appears to have distinct roles, particularly in regulating proteolysis and lipid metabolism and may influence cell differentiation. Further research is needed to fully understand the biochemical roles and cellular functions of *PCOLCE2* and to uncover its complete range of functions.

## Data availabitlity

All representative data are contained within the article. Raw data are available upon request.

## Conflict of interests

The authors declare that they have no conflicts of interest with the contents of this article.
